# COVID-19-Associated Neurological Manifestations: An Emerging Electroencephalographic Literature

**DOI:** 10.3389/fphys.2020.622466

**Published:** 2021-02-19

**Authors:** Geoffroy Vellieux, Romain Sonneville, Sérafima Vledouts, Pierre Jaquet, Anny Rouvel-Tallec, Marie-Pia d’Ortho

**Affiliations:** ^1^Université de Paris, NeuroDiderot, Inserm, Paris, France; ^2^Neurophysiologie clinique, Service de Physiologie – Explorations Fonctionnelles, AP-HP, Hôpital Bichat-Claude Bernard, Paris, France; ^3^Médecine Intensive-Réanimation, AP-HP, Hôpital Bichat-Claude Bernard, Paris, France; ^4^Université de Paris, UMR 1148, LVTS, Paris, France

**Keywords:** SARS-CoV-2, coronavirus, COVID-19, encephalopathy, neurophysiology, EEG

## Abstract

Severe acute respiratory syndrome coronavirus 2 (SARS-CoV-2) has spread worldwide since the end of year 2019 and is currently responsive for coronavirus infectious disease 2019 (COVID-19). The first reports considered COVID-19 as a respiratory tract disease responsible for pneumonia, but numerous studies rapidly emerged to warn the medical community of COVID-19-associated neurological manifestations, including encephalopathy at the acute phase and other postinfectious manifestations. Using standard visual analysis or spectral analysis, recent studies reported electroencephalographic (EEG) findings of COVID-19 patients with various neurological symptoms. Most EEG recordings were normal or revealed non-specific abnormalities, such as focal or generalized slowing, interictal epileptic figures, seizures, or status epilepticus. Interestingly, novel EEG abnormalities over frontal areas were also described at the acute phase. Underlying mechanisms leading to brain injury in COVID-19 are still unknown and matters of debate. These frontal EEG abnormalities could emphasize the hypothesis whereby SARS-CoV-2 enters the central nervous system (CNS) through olfactory structures and then spreads in CNS via frontal lobes. This hypothesis is reinforced by the presence of anosmia in a significant proportion of COVID-19 patients and by neuroimaging studies confirming orbitofrontal abnormalities. COVID-19 represents a new viral disease characterized by not only respiratory symptoms but also a systemic invasion associated with extra-respiratory signs. Neurological symptoms must be the focus of our attention, and functional brain evaluation with EEG is crucial, in combination with anatomical and functional brain imaging, to better understand its pathophysiology. Evolution of symptoms together with EEG patterns at the distance of the acute episode should also be scrutinized.

## Introduction

The coronavirus infectious disease 2019 (COVID-19), caused by the severe acute respiratory syndrome coronavirus 2 (SARS-CoV-2) infection, was initially recognized as a respiratory tract disease which could lead to an acute respiratory distress syndrome. However, there is growing evidence of a multi-organ involvement (Gupta et al., 2020). Several authors reported central nervous system (CNS) manifestations, as anosmia referring to olfactory tract involvement. Other critical presentations, including meningoencephalitis, seizures, status epilepticus (SE), encephalopathy, and altered mental status were also described (Ellul et al., 2020). Neurological complications, such as encephalopathy and seizures/SE, and electroencephalographic (EEG) abnormalities, mainly diffuse slowing and epileptiform discharges, have already been described in past viral pandemics such as influenza A H1N1 (Ekstrand et al., 2010; Kedia et al., 2011; Ibrahim and Haddad, 2014). Results of EEG in patients with COVID-19 were increasingly reported. While the volume of COVID-19-related case studies is still growing, we present the spectrum of EEG findings published at the moment, allowing physicians to be cognizant of this new and emerging literature while dealing with COVID-19 patients.

## Methods

We considered all studies with EEG findings at the acute phase in COVID-19 patients with neurological manifestations. We performed an electronic research from December 1, 2019, to October 1, 2020, using the database PUBMED by Medline with the following terms (in all fields): (i) (“EEG” OR “electroencephalogram” OR “electroencephalography”) AND (“COVID” OR “coronavirus” OR “SARS-CoV-2”) and (ii) (“brain” OR “nervous system” OR “neurology”) AND (“COVID” OR “coronavirus” OR “SARS-CoV-2”). We also scanned the reference lists of all included articles or relevant reviews for studies to be included in our work. We did not include reviews, non-English articles, unavailable full-text articles, and animal studies. After exclusion of duplicates, we screened the title/abstract or full-text reports and decided whether these met the inclusion criteria.

## EEG Observations in COVID-19 Patients

A total of 107 studies were included. Normal EEG findings were reported in adult series (Cecchetti et al., 2020; Helms et al., 2020b; Petrescu et al., 2020) and case reports of patients who displayed various neurological conditions such as focal or generalized seizures (Elgamasy et al., 2020; Fasano et al., 2020; García-Howard et al., 2020; Lyons et al., 2020), non-epileptic seizures (Logmin et al., 2020), myoclonus (Muccioli et al., 2020b; Rábano-Suárez et al., 2020), psychotic symptoms (Lim et al., 2020), encephalopathy (Andriuta et al., 2020; Chaumont et al., 2020; Delorme et al., 2020; Paterson et al., 2020; Perrin et al., 2020), encephalitis (Paterson et al., 2020), brainstem encephalitis (Khoo et al., 2020), and encephalomyelitis (Zoghi et al., 2020). Some studies also reported non-specific abnormalities without more precise EEG features specified by authors (Chougar et al., 2020; Farley and Zuberi, 2020; Freij et al., 2020; Helms et al., 2020a; Pugin et al., 2020).

### Diffuse and Focal Slowing

**FIGURE 1:**
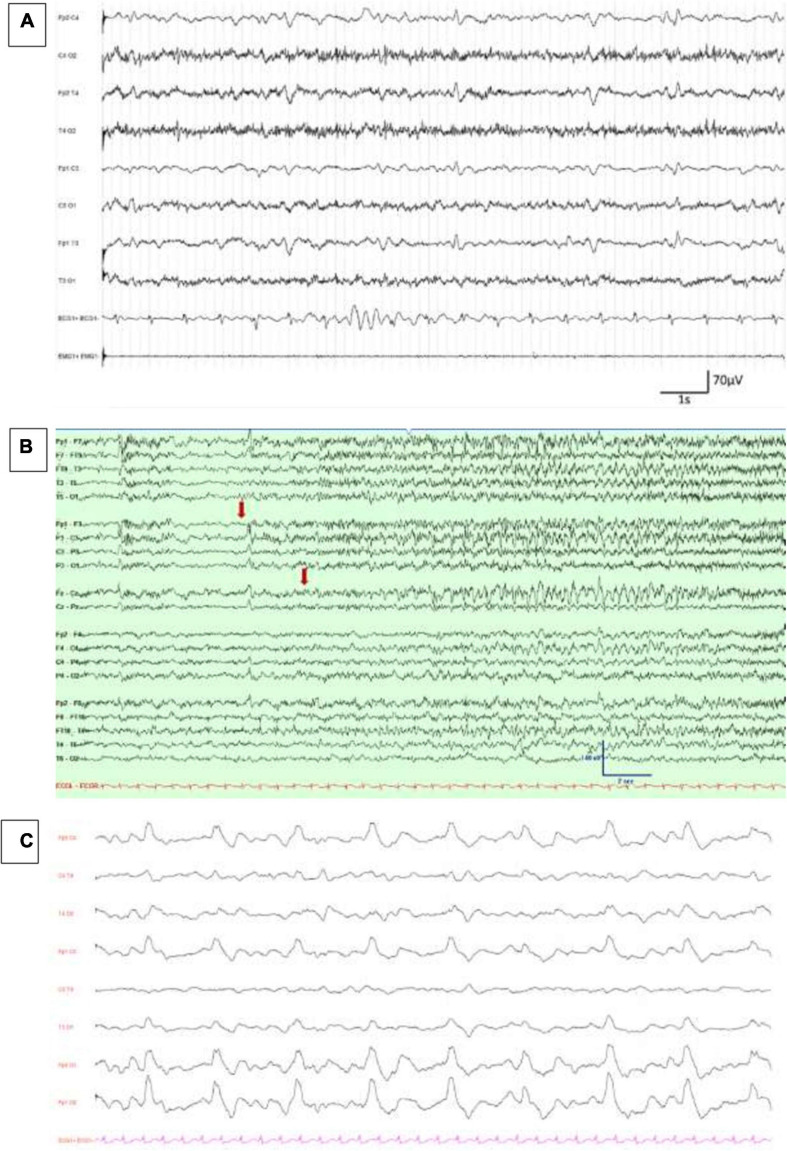
EEG findings in COVID-19 patients. **(A)** Diffuse theta–delta slowing and continuous generalized periodic discharges, reproduced with authors’ agreement from Petrescu et al. (2020). **(B)** Emergence of low-amplitude ictal fast rhythmic activity over left frontocentral and midline regions (marked with an arrow), reproduced with authors’ agreement from Somani et al. (2020). **(C)** Continuous, periodic, monomorphic diphasic, delta slow waves over both frontal areas, published in Vellieux et al. (2020).

Diffuse slowing of the background activity or focal slowing (sometimes associated with focal sharp waves or epileptiform discharges) was the most frequently published abnormality, especially in adult series (Ayub et al., 2020; Canham et al., 2020; Cecchetti et al., 2020; Chougar et al., 2020; Galanopoulou et al., 2020; Helms et al., 2020a, b; Louis et al., 2020; Pasini et al., 2020; Pellinen et al., 2020; Petrescu et al., 2020; Pilotto et al., 2020a; Scullen et al., 2020; Sethi, 2020; Vespignani et al., 2020) (Figure 1A). Main results of adult series including at least 10 patients with confirmed SARS-CoV-2 infection and EEG recordings are summarized in Table 1. Diffuse or focal slowing was also associated in many case reports with various neurological presentations, mainly of vascular or inflammatory origin. Main vascular complications included ischemic and hemorrhagic strokes (Chaumont et al., 2020; Díaz-Pérez et al., 2020; Morassi et al., 2020; Soldatelli et al., 2020; Zahid et al., 2020), intracranial hemorrhage with cerebral venous thrombosis (Roy-Gash et al., 2020), posterior reversible encephalopathy syndrome (PRES) (Llansó and Urra, 2020; Princiotta Cariddi et al., 2020), intracranial vasculitis (Dixon et al., 2020), subarachnoid hemorrhage (Harrogate et al., 2020), acute hemorrhagic leukoencephalitis or leukoencephalomyelitis (Handa et al., 2020; Kihira et al., 2020; Svedung Wettervik et al., 2020), and acute necrotizing encephalopathy (Delamarre et al., 2020; Virhammar et al., 2020). Main inflammatory syndromes included acute disseminated encephalomyelitis (ADEM) (Parsons et al., 2020; Umapathi et al., 2020), acute leukoencephalopathy (Abenza-Abildúa et al., 2020; Anand et al., 2020; Brun et al., 2020; Huang H. et al., 2020; Kihira et al., 2020; Klironomos et al., 2020), acute leukoencephalitis (Perrin et al., 2020), meningoencephalitis without any acute lesions on brain imaging (Duong et al., 2020; El-Zein et al., 2020; Pilotto et al., 2020b), Bickerstaff encephalitis (Llorente Ayuso et al., 2020), and concomitant autoimmune encephalitis (Grimaldi et al., 2020; Panariello et al., 2020). In critically ill patients, other conditions were described including post-hypoxic injury (Fischer et al., 2020; Radmanesh et al., 2020; Radnis et al., 2020; Vellieux et al., 2020), unresponsiveness after sedation discontinuation (Espinosa et al., 2020; Vellieux et al., 2020), encephalopathy or altered mental status without any acute lesions on brain imaging (Chaumont et al., 2020; Delorme et al., 2020; Filatov et al., 2020; Gaughan et al., 2020; Jang et al., 2020; Manganelli et al., 2020; Muccioli et al., 2020a; Méndez-Guerrero et al., 2020; Romero-Sánchez et al., 2020; Shekhar et al., 2020), encephalopathy with seizures (Ashraf and Sajed, 2020; Benameur et al., 2020; Farhadian et al., 2020; Haddad et al., 2020), defined toxic/metabolic encephalopathy (Flamand et al., 2020; Radmard et al., 2020; Rasmussen et al., 2020), neuroleptic malignant syndrome (Kajani et al., 2020), after seizures or SE (Anand et al., 2020; Edén et al., 2020; Emami et al., 2020), and critical illness-associated cerebral microbleeds (De Stefano et al., 2020). EEG slowing was also observed in pediatric reports (Abdel-Mannan et al., 2020; Abel et al., 2020; Dugue et al., 2020; Panda et al., 2020).

**TABLE 1:** Main results of case series including at least 10 patients admitted for COVID-19 with EEG recordings.

Series	Patients’ features	Brain imaging results	CSF results	EEG settings	Ongoing psychoactive drugs	Main EEG results
Ayub et al., 2020	>Included, *n* = 37	> CT-scan, *n* = 35 and MRI, *n* = 9	>CSF examination, *n* = 4	>Total EEG recordings, *n* = 37	>At time of EEG or the day prior	**>Background activity**
USA	M/F, *n* = 27/10	IS, *n* = 3	Abnormal WBC count, *n* = 2		Propofol, *n* = 19	Absent PDR, *n* = 34
Monocentric	Median age: 66 years	ICH, *n* = 3	Abnormal protein level, *n* = 1	>Types of EEG	Dexmedetomidine, *n* = 13	Asymmetry, *n* = 4
	Anosmia, *n* = 4			Long-term monitoring EEG, *n* = 23	Empiric AD, *n* = 11	Generalized delta and theta slowing, *n* = 34
	Intubated, *n* = 28				Midazolam or lorazepam, *n*=8	Burst suppression, *n*=5
				>Period of recordings: NA	Ketamine, *n*=2	Unreactive, *n*=1
	*Prior neurological history*					
	Stroke, *n* = 6			>EEG indication		**>Rhythmic and periodic patterns**
	Cerebral aneurysm, *n* = 1			Altered mental status, *n*=24		GPDs without triphasic waves, *n*=4
	Epilepsy, *n* = 1			Possible seizures, *n*=11		GPDs with triphasic waves, *n*=8
	ICH, *n* = 1			Cardiac arrest, *n*=2		SIRPIDs, *n*=3
	DLB, *n* = 1					GRDA, *n*=5
						LRDA, *n*=1
						**>Epileptiform findings and seizures**
						Burst suppression with epileptiform activity, *n*=4
						Focal sporadic discharges, *n*=1
						Multifocal sporadic discharges, *n*=6
						Generalized sporadic discharges without triphasic waves, *n*=8
						Generalized NCSE, *n*=1

Canham et al., 2020	**>**Included, *n*=10	**>**CT-scan, *n*=10	**>**CSF examination, *n*=6	**>**Total EEG recordings, *n*=11	**>**At time of EEG	**>Background activity**
United Kingdom	M/F, *n*=8/2	Normal, *n*=3	Abnormal WBC count, *n*=3		Levetiracetam, *n*=6	Generalized symmetrical slowing, *n*=11
Multicentric	Median age: 65 years	Small vessel disease, *n*=4	Abnormal protein level, *n*=4	**>**Types of EEG	Propofol, *n*=2	Anterior emphasis of slow activity, *n*=3
	Anosmia/agueusia: NA	SAH, *n*=2	Negative HSV 1&2, VZV	9 electrodes 20-30 min EEG, *n*=11	Alfentanil, *n*=2	Asymmetry, *n*=1
	Intubated: NA	Atrophy, *n*=2	and enterovirus PCR, *n*=6		Phenytoin, *n*=2	
			Negative SARS-CoV-2 PCR, *n*=2	**>**Period of recordings: NA	Valproate, *n*=2	**>Rhythmic and periodic patterns**
	*Prior neurological history*	**>**MRI, *n*=4			Lamotrigine, *n*=1	FIRDA, *n*=1
	SAH, *n*=1	Normal, *n*=1		**>**EEG indication	Gabapentin, *n*=1	
	Stroke, *n*=1	Small vessel disease, *n*=2		Altered mental status, *n*=6	Carabamazepine, *n*=1	**>Epileptiform findings and seizures, *n*=0**
	Learning difficulties, *n*=1	IS, *n*=1		Seizure, *n*=6	Lacosamide, *n*=1	
	Essential tremor, *n*=1	Atrophy, *n*=1		Delirium, *n*=2	Primidone, *n*=1	
	Epilepsy, *n*=1				Amitriptyline, *n*=1	
					Lorazepam, *n*=1	
					Citalopram, *n*=1	
					Olanzapine, *n*=1	
					Clozapine, *n*=1	
					Paliperidone, *n*=1	
					Midazolam, *n*=1	
					Remifentanyl, *n*=1	
					Morphine, *n*=1	

Cecchetti et al., 2020	**>**Included, *n*=18	**>**CT-scan and/or MRI	**>**CSF examination, *n* = 1	**>**Total EEG recordings, *n*=18	NA	**>Background activity**
Italy	M/F, *n*=11/7	PRES, *n*=1	Normal WBC count, *n*=1			Normal or with mild alteration, *n*=5
Monocentric	Mean age: 67 years	Remote ICH, *n*=1	Normal protein level, *n*=1	**>**Types of EEG		With moderate alteration, *n*=9
	Anosmia/agueusia, *n*=0	Remote IS, *n*=1	Negative bacteriologic and	Basal EEG, *n*=18		With severe alteration, *n*=4
	Intubated: NA	Glioblastoma, *n*=1	virologic assays (including			Generalized slowing, *n*=16
		Metastasis, *n*=1	SARS-CoV-2 RT-PCR), *n*=1	**>**Period of recordings: NA		Anterior (bifrontal) prevalence of slow waves, *n*=10
	*Prior neurological history*	Traumatic SDH, *n*=1				Focal slowing, *n*=7
	NA	Anterior pontine demyelinating lesion, *n*=1		**>**EEG indication		
				Transient loss of consciousness, *n*=5		**>Rhythmic and periodic patterns, *n*=0**
				Seizures/spasms, *n*=5		
				Coma, *n*=5		**>Epileptiform findings and seizures**
				Delirium, *n*=3		Epileptiform discharges, *n*=2
						Seizures, *n*=0
Chougar et al., 2020	**>**Included, *n*=73	**>**MRI, *n*=73	**>**CSF examination, *n*=39	**>**Total EEG recordings, *n*=40	NA	**>Background activity / Epileptiform findings and seizures**
France	M/F, *n*=48/25	No significant abnormalities, *n*=30	Abnormal WBC count, *n*=8			Pathological findings related to seizure or encephalopathy, *n*=9
Monocentric	Mean age: 56 years	Acute IS, *n*=17	Abnormal protein level, *n*=10	**>**Types of EEG: NA		Non-specific findings, *n*=24
	Anosmia/agueusia, *n*=4	Multiple microhemorrhages, *n*=8	Oligoclonal bands, *n*=2			
	Intubated: NA	Multifocal enhancing WM lesions, *n*=4	Negative bacteriologic and	**>**Period of recordings: NA		**>Rhythmic and periodic patterns: NA**
		Basal ganglia lesions, *n*=4	virologic assays (including			
	*Prior neurological history*	Hypoxic-ischemic lesions, *n*=3	HSV 1&2, VZV, CMV, EBV	**>**EEG indication: NA		
	Stroke, *n*=NA	Cytotoxic lesions of the CC, *n*=3	and SARS-CoV-2 RT-PCR), *n*=39			
		Central pontine myelinolysis, *n*=3				
		PRES, *n*=2				
		Meningeal enhancement, *n*=2				
		Neuritis, *n*=2				
		Deep venous thrombosis, *n*=1				
		Corticospinal tracts FLAIR hyperintensity, *n*=1				
		**>**Perfusion MRI, *n*=46				
		Seizure-related perfusion abnormalities, *n*=9				
		Recent or old vascular lesions-related perfusion abnormalities, *n*=4				
		Perfusion abnormalities unrelated to seizures or ischemia, *n*=10				

Galanopoulou et al., 2020	**>**Included, *n*=22	**>**Modality: NA (at least 1 brain MRI)	NA	**>**Total EEG recordings, *n*=31	**>**During hospital stay (at time of EEG: NA)	**>Background activity**
USA	M/F, *n*=14/8 Mean age: 63 years Anosmia/agueusia: NA Intubated, *n*=14	Subcortical and mild periventricular WM signal			Sedatives, *n*=14	Bilateral slowing, *n*=22 Focal slowing, *n*=5 Asymmetry, *n*=3 Absent PDR, *n*=18 Slow PDR, *n*=4 Discontinuous or burst suppression, *n*=1
Multicentric		hyperintensity, *n* = 1		**>**Types of EEG	AD, *n*=12	
		SAH due to aneurysm, *n*=1		10 electrodes/8-channel EEG, *n*=20		
		SDH, *n*=1		Routine EEG, *n*=4		
				cEEG, *n*=7		
	*Prior neurological history*					
	Epilepsy, *n*=4			**>**Period of recordings: NA		
	Neurological disorders except epilepsy, *n*=7					**>Rhythmic and periodic patterns**
				**>**EEG indication		Generalized or frontal RDA, *n*=3
				Altered mental status, *n*=20		Temporal LRDA, *n*=1
				Motor seizure-like event or seizure		Bifrontal sharply contoured periodic waves, *n*=1
				at presentation or confusion		
				resembling prior seizures, *n*=12		**>Epileptiform findings and seizures**
				Gaze deviation, *n*=2		Bilateral frontal sharp waves, *n*=6
				Confusion at presentation and no		Unilateral frontal sharp waves, *n*=2
				prior seizures, *n*=1		Temporal or hemispheric sharp waves, *n*=2
						Seizures, *n*=0

Helms et al., 2020a	**>**Included, *n*=58	**>**MRI, *n*=13	**>**CSF examination, *n*=7	**>**Total EEG recordings, *n*=8	**>**During hospital stay (at time of EEG: NA)	**>Background activity**
France	M/F: NA	Leptomeningeal enhancement, *n*=8	Normal WBC count, *n*=7		Sufentanil, *n*=58	Nonspecific changes, *n*=8
Bicentric	Median age: 63 years	Acute IS, *n*=2	Elevated protein level, *n*=1	**>**Types of EEG: NA	Midazolam, *n*=50	Diffuse bifrontal slowing, *n*=1
	Anosmia/agueusia: NA Intubated, *n*=58	Subacute IS, *n*=1	Oligoclonal bands with mirror pattern, *n*=2	**>**Period of recordings: NA	Propofol, *n*=27	**>Rhythmic and periodic patterns: NA**
		**>**Perfusion MRI, *n*=11	Negative SARS-CoV-2 RT-PCR, *n*=7	**>**EEG indication: NA		
	*Prior neurological history*	Bilateral frontotemporal hypoperfusion, *n*=11				**>Epileptiform findings and seizures: NA**
	TIA, partial epilepsy, MCI, *n*=7					
Helms et al., 2020b	**>**Included, *n*=140	**>**MRI, *n*=28	**>**CSF examination, *n*=25	**>**Total EEG recordings, *n*=42	>During hospital stay (at time of EEG: NA)	**>Background activity**
France	M/F, *n*=100/40	Subarachnoid spaces FLAIR and T1 contrast	Elevated WBC count, *n*=3		Midazolam, *n*=121	Normal, *n*=5
Bicentric	Median age: 62 years Anosmia/agueusia: NA Intubated, *n*=140 *Prior neurological history* Stroke/TIA, *n=9* Migraine, *n=5* Mild cognitive alteration, *n*=4 Partial epilepsy, *n*=2 Trauma brain injury, *n*=2 Aneurysm, *n*=1	enhancement, *n*=17 WM microhemorrhages, *n*=7 WM FLAIR hyperintensities, *n*=4, with small foci of contrast enhancement, *n*=2 and diffusion hyperintensities, *n*=2 Acute IS, *n*=2 Intraparenchymal hematoma, n=1 Preexisting IS, n=1 **>**Perfusion MRI, *n*=26 Perfusion abnormalities, *n*=17	Elevated protein level, *n*=8 Elevated IgG levels, *n*=9 Oligoclonal bands with mirror pattern, *n*=13 Positive SARS-CoV-2 RT-PCR (negative result in blood), *n*=1 Negative bacterial cultures and viral research (HSV 1&2, enterovirus), *n = 25*	**>**Types of EEG: NA **>**Period of recordings: NA > EEG indication Unexplained and persistent alteredconsciousness after prolongedsedation discontinuation (> 3 days) Multimodality neurologicalscreening in combination with brainMRI and/or CSF examination	Sufentanil, *n*=138 Propofol, *n*=83	Unspecific abnormalities, with low voltage, rapid rhythm, and lack of asymmetry, *n*=26 Diffuse, especially bifrontal, slow activity *n*=11 **>Rhythmic and periodic patterns: NA > Epileptiform findings and seizures: NA**

Louis et al., 2020	>Included, *n*=22	**>**CT-scan, *n*=18	NA	**>**Total EEG recordings, *n*=22 **>**Types of EEG cEEG, *n*=19 Routine EEG, *n*=3	>At time of EEG	**>Background activity**
USA Monocentric	M/F, *n*=14/8	Possible IS, *n*=2			Sedative drugs (including fentanyl, propofol and/or midazolam), *n*=14	Continuous generalized polymorphic delta slowing, *n*=19
	Mean age: 67 years	Acute IS, *n*=1				Slow PDR, *n*=9
	Anosmia/agueusia: NA	ICH, *n*=1				Absent PDR, *n*=11
	Intubated, *n*=18					Normal PDR, *n*=2
		**>**MRI, *n*=1				
	*Prior neurological history*	Acute IS, *n*=1		**>**Period of recordings: NA		**>Rhythmic and periodic patterns**
	Epilepsy, *n*=2					GPDs, *n*=7
	Stroke, *n*=1			**>**EEG indication		GPDs with triphasic morphology, *n*=5
	Headache, *n*=1			Altered mental status, *n*=17		GPDs with sharply contoured morphology, *n*=2
	Traumatic brain injury, *n*=1			Seizure-like event, *n*=5		Intermittent GRDA, *n*=11
	Spinal stenosis, *n*=1					Hemispheric LRDA, *n*=1
						**>Epileptiform findings and seizures**
						Epileptic abnormalities, *n*=5
						Seizures, *n*=2

Pasini et al., 2020	>Included, *n*=15	**>**CT-scan, *n*=8	**>**CSF examination, *n*=5	>Total EEG recordings, *n*=15	NA	*Subset of non post-anoxic patients, n=13*
Italy	M/F, *n*=6/9	Normal, *n*=8	Elevated protein level, *n*=1			**>Background activity**
Monocentric	Mean age: 65 years		Negative SARS-CoV-2 detection, *n*=5	**>**Types of EEG		Generalized slowing with theta prevalence, *n*=5
	Anosmia/agueusia: NA	**>**MRI, *n*=6		18 electrodes EEG, *n*=15		Generalized slowing with intrusions of theta/delta activity, *n*=4
	Intubated: NA	Mild WM T2 hyperintensity, *n*=2		>Period of recordings: NA		Focal slowing predominantly over the frontal or central regions *n*=3
						Unreactive, *n*=10
	*Prior neurological history*					
	Cognitive decline, *n*=2			**>**EEG indication		**>Rhythmic and periodic patterns**
	Limbic encephalitis, *n*=1			Confusion, *n*=11		FIRDA, *n*=1
	Frontal metastasis, *n*=1			Impairment of consciousness, *n*=4 with post-anoxic coma, *n*=2		**>Epileptiform findings and seizures**
				Aphasia, *n* = 1		Epileptiform abnormalities, *n*=0
						*Subset of post-anoxic comas, n=2*
						Severely suppressed activity, *n*=1
						Discontinued activity compatible with post-anoxic SE, *n*=1
						Unreactive, *n*=2
Pellinen et al., 2020 USA Multicentric	**>**Included, *n*=111 M/F, *n*=79/32 Median age: 64 years Anosmia/agueusia: NA Intubated, *n*=79 *Prior neurological history* Stroke, *n*=23 Epilepsy, *n*=13 ICH, *n*=4 Dementia, *n*=4 Developmental delay/intellectual disability, *n*=3 Brain tumor, *n*=3 Traumatic brain injury, *n*=2 Parkinson disease, *n*=2 Vascular malformation, *n*=1 Tuberous sclerosis complex, *n*=1 Herpes encephalitis, *n*=1	>Brain imaging, *n*=90 (with CT-scan only, *n*=75) Acute IS, *n*=18 Acute ICH, *n*=15 Cerebral edema, *n*=6 Diffuse leukoencephalopathy with microhemorrhages, *n* = 4 Mixed acute ischemic and hemorrhagic lesions, *n*=3	NA	**>**Total EEG recordings, *n*=118 **>**Types of EEG 21-channel cEEG for a target of at least 24 hours, *n*=111 Rapid EEG system with 8-bipolar channel montage 0.5-12 hours, *n*=7 >Period of recordings: NA **>**EEG indication Persistent encephalopathy, *n*=72 Paroxysmal activity of unclear cause, *n*=25 Seizure exacerbation, *n*=10 Cardiac arrest *n*=11	**>**During EEG Sedative drugs (including propofol, midazolam, pentobarbital, dexmedetomidine and/or fentanyl) *n*=67 **>**Prior to EEG AD, *n*=57	**>Background activity** Normal, *n*=5 Mild generalized slowing, *n*=17 Moderate generalized slowing, *n*=60 Severe generalized slowing/discontinuous/ECI, *n*=29 Focal slowing, *n*=27 **>Rhythmic and periodic patterns** GRDA, *n*=4 LRDA, *n*=7 LRDA and GRDA, *n*=2 GPDs, *n*=11 LPDs, *n*=3 **>Epileptiform findings and seizures** Focal epileptiform discharges, *n*=12 Multifocal epileptiform discharges, *n*=6 Generalized epileptiform discharges, *n*=5 Seizures, *n*=8 NCSE, *n*=2

Petrescu et al., 2020	**>**Included, *n*=36	**>**CT-scan, *n*=14	>CSF examination, *n*=4	**>**Total EEG recordings, *n*=40	**>**At time of EEG	**>Background activity**
France	M/F, *n*=26/10	Normal, *n*=4	Normal, *n*=4		Levetiracetam, *n*=6	Normal, *n*=4
Monocentric	Mean age: 70 years	Atrophy, *n*=9		>Types of EEG	Sedations, *n*=5	Mildly altered, *n*=19
	Anosmia/agueusia: NA	IS, *n*=2		Routine 20 min EEG, *n*=40	Risperidone, *n*=4	Moderately altered, *n*=4
	Intubated, *n*=11	Calcification, *n*=2			Clobazam, *n*=2	Severely altered, *n*=8
		SDH, *n*=1		**>**Period of recordings: NA	Dexmedetomidine, *n*=2	Critically altered, *n*=5
	*Prior neurological history*	Leukoaraiosis, *n*=1			Citalopram or escitalopram, *n*=2	Focal bioccipital slowing, *n*=1
	Dementia, *n*=10	Meningioma, *n*=1		**>**EEG indication	Midazolam, *n*=2	Sporadic triphasic waves, *n*=1
	Stroke, *n*=3	Postoperative lesion, *n*=1		Fluctuating alertness, *n*=13	Oxazepam, *n*=2	
	SDH, *n*=2			Confusion, *n*=9	Morphine, *n*=2	**>Rhythmic and periodic patterns**
	Memory impairment, *n*=1	**>**MRI, *n*=11		Delayed awakening after stopping	Oxazepine, *n*=1	RDA, *n*=7 with frontal predominant, *n*=1
	Hydrocephalus, *n*=1	Atrophy, *n*=4		sedation or inadequate emerge of	Haloperidol, *n*=1	GPDs, *n*=6
	Epilepsy, *n*=1	IS, *n*=2		sedation, *n*=8	Doxylamine succinate, *n*=1	Multifocal PDs, *n*=2
	Parkinson disease, *n*=1	SDH, *n*=2		Focal neurologic symptoms, *n*=6	Lacosamide, *n*=1	
		Gliosis of CC, *n*=1		Seizures, *n*=3	Diazepam, *n*=1	**>Epileptiform findings and seizures**
		Leukoaraiosis, *n*=1		Abnormal movements, *n*=3	Valproate, *n*=1	Epileptiform discharges, *n*=0
		Leptomeningeal enhancement, *n*=1		Cardiac arrest, *n*=1	Bromazepam, *n*=1	Seizures, *n*=0
		Probable septic lesions (multiple ischemic and hemorrhagic lesions) related to endocarditis, *n*=1		Encephalopathy, *n*=1	Gabapentin, *n*=1	
				Control follow-up, *n*=1	Paroxetine, *n*=1	
		Multiple FLAIR hyperintense lesions, *n*=1			Alprazolam, *n*=1	
					Hydroxyzine, *n*=1	
					Mianserine, *n*=1	
Pilotto et al., 2020a Italy Multicentric	>Included patients, *n*=25 M/F, *n*=15/10 Mean age: 66 years Anosmia/agueusia: NA Intubated, *n*=4 *Prior neurological history* Stroke, *n*=2 Mental retardation, *n*=1 Possible encephalitis and Behçet disease, *n*=1	>MRI, *n* = 25 Normal, *n*=13 Multiple subcortical T2-hyperintensities, *n*=4 Focal cortical T2 and DWI hyperintensities, *n*=3 Acute necrotizing encephalopathy, *n*=2 Limbic encephalitis, *n*=2 ADEM, *n*=1 Leptomeningeal enhancement, *n*=1	**>**CSF examination, *n*=25 Normal, *n*=8 Elevated WBC count, *n*=9 Elevated protein level, *n*=15 Negative bacteriological and virological screening, *n*=25 Negative SARS-CoV-2 RT-PCR, *n*=14	**>**Total EEG recordings, *n*=25 **>**Types of EEG: NA **>**Period of recordings: NA **>**EEG indication Delirium/altered mental status, *n*=17 Aphasia/dysarthria, *n*=6 Seizures, *n*=6	NA	**>Background activity** Generalized slowing especially localized to frontal derivations, *n*=16 **>Rhythmic and periodic patterns: NA >Epileptiform findings and seizures** Focal epileptic alterations, *n*=6

Scullen et al., 2020	>Included patients, *n*=27	**>**CT-scan, *n*=27	NA	**>**Total EEG recordings, *n*=13	NA	**>Background activity**
USA Monocentric	M/F, *n*=14/13	Focal hypodensities in deep structures, *n*=14				Generalized encephalopathy (i.e. irregular slowing with delta and theta frequency oscillations), *n*=11
	Mean age: 60 years	Diffuse hypoattenuation, *n*=6		**>**Types of EEG		
	Anosmia/agueusia, *n*=1	Subacute IS, *n*=4		cEEG, *n*=13		**>Rhythmic and periodic patterns: NA**
	Intubated: NA	Subcortical parenchymal hematoma, *n*=3				
				>Period of recordings: NA		**>Epileptiform findings and seizures**
	*Prior neurological history*	**>**MRI, *n*=8		**>**EEG indication		NCSE, *n*=1
	Stroke, *n*=3	Viral encephalitis with diffuse involvement of the		Pronounced encephalopathy not explained by previous CT alone, *n*=9		
	Pseudotumor cerebri, *n*=1	deep WM, CC and basal ganglia, *n*=NA		Pronounced encephalopathy not explained by previous combined CT and MRI, *n*=4		

Sethi, 2020	NA	NA	NA	**>**Total EEG recordings, *n*=20	NA	**>Background activity**
USA						Diffuse theta and delta slowing
Monocentric				**>**Types of EEG: NA		
						**>Rhythmic and periodic patterns: NA**
				**>**Period of recordings: NA		
						**>Epileptiform findings and seizures: NA**
				**>**EEG indication		
				Altered mental status		

Vespignani et al., 2020	**>**Included patients, *n*=26	**>**CT-scan, *n*=1	**>**CSF examination, *n*=2	>Total EEG recordings, *n*=26	*Subset of the 5 patients with PDs*	**>Background activity**
France		Occipital cyst, *n*=1	Normal, *n*=2		**>**At time of EEG	Diffuse slowing without PDs, *n*=19
Multicentric	*Subset of patients with PDs, n=5*			**>**Types of EEG	Propofol, *n*=2	Isoelectric, *n*=2
	M/F, *n*=4/1	**>**MRI, *n*=1		9 electrodes 30 min EEG, *n*=26	Fentanyl, *n*=2	
	Mean age: 67 years	Diffuse WM hyperintensities, *n*=1			Midazolam, *n*=1	**>Rhythmic and periodic patterns**
	Anosmia/agueusia: NA			**>**Period of recordings: NA		GPDs with frontal involvement, *n*=4
	Intubated, *n*=4					LPDs with frontal involvement, *n*=1
	Prior neurological history: NA			**>**EEG indication		
				Mental status changes		**>Epileptiform findings and seizures, *n*=0**
				Poor responsiveness		
				Determine the presence of SE in non-arousable patients		
				*Subset of the 5 patients with PDs*		
				Poor or absent responsiveness, *n*=4		
				Cardiac arrest, *n*=1		
				Confusion and lethargy, *n*=1		

*AD: antiepileptic drug, ADEM: acute disseminated encephalomyelitis, CC: corpus callosum, cEEG: continuous EEG, CMV: cytomegalovirus, CSF: cerebrospinal fluid, CT: computed tomography, DLB: dementia with Lewy bodies, DWI: diffusion weighted imaging, EBV: Epstein-Barr virus, ECI: electrocerebral inactivity, EEG: electroencephalogram, FIRDA: frontal intermittent rhythmic delta activity, FLAIR: fluid-attenuated inversion recovery, GPDs: generalized periodic discharges, GRDA: generalized rhythmic delta activity, HSV: herpes simplex virus, ICH: intracranial hemorrhage, IS: ischemic stroke, LRDA: lateralized rhythmic delta activity, MCI: mild cognitive impairment, M/F: male/female, n: number, MRI: magnetic resonance imaging, NA: not available, NCSE: non convulsive SE, PCR: polymerase chain reaction, PDR: posterior dominant rhythm, PDs: periodic discharges, PRES: posterior reversible encephalopathy syndrome, RDA: rhythmic delta activity, RT-PCR: reverse transcriptase PCR, SAH: subarachnoid hemorrhage, SARS-CoV-2: severe acute respiratory syndrome coronavirus 2, SDH: subdural hematoma, SE: status epilepticus, SIRPIDs: stimulus-induced rhythmic, periodic or ictal discharges, TIA: transient ischemic attack, VZV: varicella-zoster virus, WBC: white blood cells, WM: white matter.*

### Seizures and SE

Seizures and/or SE were recorded in 10 patients out of 111 included in the series of Pellinen et al. (2020), in 2 out of 22 in the series of Louis et al. (2020), in 1 out of 37 in the series of Ayub et al. (2020), in 1 out of 15 in the series of Pasini et al. (2020), in 1 out of 27 in the series of Scullen et al. (2020), and in an unknown precise number of patients out of the 73 included in the series of Chougar et al. (2020) (Table 1).

Seizures and/or SE were recorded in reports of patients without any acute or chronic cortical lesions on brain imaging nor cerebrospinal fluid (CSF) abnormalities. The EEG of the patient reported by Balloy et al. (2020) revealed two widespread, but predominantly in frontal localizations, seizures that were interrupted by a moderate interictal frontal activity. Sohal and Mansur (2020) reported a patient whose 24-h EEG revealed six left temporal seizures and left temporal sharp waves. One of the two patients reported by Somani et al. (2020) displayed, on a continuous EEG (cEEG) monitoring, multiple seizures emanating from the midline and left frontocentral regions (Figure 1B). Hepburn et al. (2020) reported the cases of two patients whose cEEG monitoring showed, for the first one, three focal seizures arising from the right frontocentral region and, for the second one, left more than right frontotemporal seizures which progressed to focal SE. The EEG of the patient reported by Le Guennec et al. (2020) revealed a non-convulsive SE (NCSE) over the right frontal region. The brain MRI of this patient only showed peri-ictal diffusion abnormalities over the right orbital and mesial prefrontal cortex and right caudate nucleus. Flamand et al. (2020) reported the case of a patient who benefited from several EEG. The first two EEG findings were consistent with a bilateral frontal SE. One EEG in the series of five patients reported by Chen et al. (2020) showed a bifrontal SE, and another one revealed a generalized NCSE. Finally, the EEG of the patient reported by Rodrigo-Armenteros et al. (2020) showed a bilateral frontotemporal NCSE.

Seizures and/or SE were recorded more rarely in patients with acute CNS lesions on brain imaging and/or significant CSF abnormalities, of either vascular or inflammatory origin. Among the four patients with a PRES reported by Parauda et al. (2020), two had seizures or SE emanating from posterior regions: for the first one, a focal NCSE arising from the left posterior quadrant and, for the second one, focal seizures arising from the right posterior quadrant. The history of a 2-month-old boy was published by Schupper et al. (2020). His brain imaging revealed multiple infarctions with hemorrhagic transformations, and his cEEG showed NCSE. Zanin et al. (2020) published the case of a patient with diffuse CNS demyelinating lesions on brain and spine imaging whose EEG revealed two seizures starting from the right frontotemporal region and diffusing in the homologous contralateral hemisphere. Hussein et al. (2020) reported the case of a patient with an ADEM whose EEG revealed left hemispheric seizures and, 3 days later, brief focal right posterior seizures. Finally, Bernard-Valnet et al. (2020) reported the history of a patient with a lymphocytic meningitis on CSF analysis with normal brain MRI whose EEG showed a focal anterior NCSE.

Seizures and/or SE were recorded in patients with a prior neurological history and radiological sequelae but without any acute lesions. The EEG of the second patient, who had a prior history of skull base surgery, reported by Somani et al. (2020) showed recurrent seizures emanating from either right or left frontocentroparietal regions. Vollono et al. (2020) reported the case of a left frontocentrotemporal SE in a patient with a remote *herpes simplex virus* 1 encephalitis.

Seizures were reported on cEEG in the series of 33 patients published by Radmard et al. (2020), as frontotemporal and parasagittal seizures in two patients, but without precise imaging or CSF results available for these two patients.

### Rhythmic and Periodic Discharges

Rhythmic discharges were mentioned in series, as generalized rhythmic delta activity (GRDA) (Ayub et al., 2020; Galanopoulou et al., 2020; Louis et al., 2020; Pellinen et al., 2020; Petrescu et al., 2020), lateralized rhythmic delta activity (LRDA) (Ayub et al., 2020; Galanopoulou et al., 2020; Louis et al., 2020; Pellinen et al., 2020), and frontal intermittent rhythmic delta activity (Canham et al., 2020; Pasini et al., 2020) (Table 1). Rhythmic patterns were also reported in a few case reports. Vandervorst et al. (2020) published the EEG of a patient with a clinical and radiological picture of encephalitis with temporal bilateral more left than right imaging abnormalities. The EEG showed short-lasting left temporal LRDA. In the series of Beach et al. (2020), one patient, with a previous history of dementia with Lewy bodies and remote traumatic brain injury, displayed GRDA with sharp contouring and bifrontal predominance, without any acute lesions on brain imaging. The EEG of the three other patients reported in the series of Chen et al. (2020) previously mentioned revealed GRDA, with unremarkable CSF analysis for the three and no acute lesions on brain imaging for one of them (unavailable for the two others). One EEG recorded among the seven patients reported by Anand et al. (2020) showed GRDA in a patient with extensive leukoencephalopathy on brain MRI and normal CSF sample.

Periodic discharges were noted in series, as generalized periodic discharges (GPDs) (Ayub et al., 2020; Galanopoulou et al., 2020; Louis et al., 2020; Pellinen et al., 2020; Petrescu et al., 2020; Vespignani et al., 2020) and lateralized periodic discharges (LPDs) (Pellinen et al., 2020; Petrescu et al., 2020; Vespignani et al., 2020) (Table 1). Especially, in the series of Vespignani et al. (2020), five EEGs out of 26 showed periodic discharges. Four of these five patients were under mechanical ventilation (MV), and three were sedated. One patient suffered from a cardiac arrest. EEG showed periodic (with a < 4 s interval), monomorphic biphasic, delta activity, which was diffuse with frontal predominance for four and lateralized over right frontal area for one. The second patient reported in the work of Beach al. previously mentioned presented with a left-sided acute-on-chronic subdural hematoma (SDH) due to a fall with head trauma. The EEG showed frequent runs of epileptiform GPDs (Beach et al., 2020). Young et al. (2020) reported 1–1.5 Hz LPDs and diffuse delta–theta slowing in a patient who displayed Creutzfeldt–Jakob disease in tandem with symptomatic onset of COVID-19. Conte et al. (2020) published the history of a patient who presented a severe COVID-19 pneumonia and then a PRES-like encephalopathy. She displayed focal seizures, and after seizure treatment, EEG revealed LPDs in the right posterior regions. Vellieux et al. (2020) published the EEG of two critically ill patients who displayed a severe COVID-19 pneumonia requiring MV. For the first one, the brain MRI was consistent with a hypoxic encephalopathy, and the EEG was recorded while he was sedated and under extracorporeal membrane oxygenation. For the second one, the EEG was recorded 24 h after sedation discontinuation. EEG revealed continuous, symmetric, non-reactive, generalized but mainly bifrontal, monomorphic diphasic or even triphasic, periodic (with a short interval of 1–2 s) delta slow waves (Figure 1C). One patient, without any acute abnormalities on brain MRI and with normal CSF analysis, reported by Delorme et al. (2020) showed GPDs. In the previously mentioned case reported by Le Guennec et al. (2020), a control follow-up EEG was recorded the day after the first EEG. It showed persistent right frontal LPDs with a short interval (0.7–1.2 s). The brain MRI performed 1 month later was normal. Finally, the previously mentioned patient reported by Flamand et al. (2020) who benefited from iterative EEG showed, on the last two recordings, a generalized periodic triphasic activity with short periods (1–1.5 s) over a worsened background activity, without concomitant metabolic disorders.

### Spectral Analysis

Two studies reported quantitative analysis of EEG (qEEG) in COVID-19 patients. The study of Pastor et al. (2020) reported 20 patients with COVID-19 encephalopathy for whom standard visual analysis of EEG showed scarce abnormalities. However, compared to 31 infectious toxic encephalopathy patients and 21 post-cardiorespiratory arrest encephalopathy patients, some qEEG features were specific in COVID-19 patients, such as the distribution of EEG bands, the structure of Shannon’s spectral entropy, and the hemispheric connectivity. Finally, the study of Pati et al. (2020) showed that some qEEG markers, especially an increase in both the theta power and its temporal variance during EEG reactivity, can predict a good neurological outcome in 10 critically ill COVID-19 patients.

## Discussion

The vast majority of these studies emphasized the absence of specificity of EEG abnormalities reported in COVID-19 patients, as generalized slowing of the background activity, focal slowing sometimes associated with sharp waves, seizures, SE, and predictable pattern of metabolic/toxic or postanoxic encephalopathy in ICU patients. Numerous EEGs in the context of COVID-19 were recorded in elderly patients and mainly in male patients, with multiple comorbidities especially chronic brain disorders, under various psychotropic drugs or in critically ill conditions. Confounding factors such as infections, metabolic disturbances, severe hypoxemia, hyperthermia, and psychotropic drugs (such as antiepileptic or sedative drugs) were frequent at the time of EEG recordings. All these confounding factors may contribute to the modification of brain activity and therefore EEG findings. Thus, based on the current literature, it seems not possible to identify a specific EEG pattern due to the suspected neuroinvasion of SARS-CoV-2 in patients who displayed neurological manifestations of COVID-19.

Most current studies with available EEG data are case reports or retrospective single-center series. All reported patients are very heterogeneous concerning prior neurological histories, illness severity, and use of psychotropic drugs. Moreover, some studies reported EEG recorded with limited montage and number of electrodes that may limit the detection of EEG abnormalities. EEG is not a systematic exam in the diagnostic workup of COVID-19 patients. All patients reported in the current literature had an EEG for an urgent clinical indication due to concerning neurological symptoms. A wider neurological multimodality screening, including EEG, of COVID-19 patients may be suggested to grow the body of knowledge on the SARS-CoV-2 infection. However, it will face many logistic difficulties and ethical and safety concerns regarding the availability of trained personnel to EEG recordings and the risk of contamination with the SARS-CoV-2.

It should be pointed out that many EEG abnormalities reported were recorded over anterior or frontal regions. Regardless of EEG montage used by clinicians and neurophysiologists, it thus seems essential to include frontal electrodes. Periodicity, morphology, and reactivity of these frontal abnormalities were not mentioned in all studies. Moreover, a few reported periodic patterns, as GPDs (Ayub et al., 2020; Beach et al., 2020; Delorme et al., 2020; Louis et al., 2020; Pellinen et al., 2020; Petrescu et al., 2020), GPDs with bifrontal predominance (Galanopoulou et al., 2020; Vellieux et al., 2020; Vespignani et al., 2020), and LPDs (Conte et al., 2020; Le Guennec et al., 2020; Pellinen et al., 2020; Petrescu et al., 2020; Vespignani et al., 2020; Young et al., 2020). In particular, these frontal periodic discharges were monomorphic and displayed a short interval, and the absence of reactivity was noted (Vellieux et al., 2020; Vespignani et al., 2020). These frontal periodic discharges may indicate an acute neurological process linked to the brain SARS-CoV-2 infection. In COVID-19 patients, the combination of the frontal localization of these EEG discharges, the frequently reported anosmia (Yazdanpanah et al., 2020), the olfactory bulb abnormalities found on brain imaging (Lin et al., 2020), and the hypometabolism within the orbitofrontal cortex on functional brain imaging (Karimi-Galougahi et al., 2020) may support the hypothesis whereby SARS-CoV-2 could invade the brain through the olfactory pathway. Then, it could spread transneuronally to other related brain areas particularly to frontal lobes, especially the orbital prefrontal cortex, which are adjacent to olfactory structures (Huang J. et al., 2020).

## Conclusion

In the context of the SARS-CoV-2 infection, increasing EEG results were published along with clinical reports describing various neurological symptoms in patients with COVID-19. Due to the suspected neuroinvasion of SARS-CoV-2, the major issue when interpreting EEG is to determine whether the observed abnormalities reflect this viral neuroinvasion, a severe encephalopathy with systemic and brain inflammation, hypoxemia and hyperthermia, and/or many confounding factors especially due to critical illness. At this time, no study had described specific EEG abnormalities of the SARS-CoV-2 infection. The majority of currently reported EEGs showed generalized slowing, focal slowing, epileptiform discharges with seizures, and SE. However, frontal discharges, for some periodic, may integrate in the olfactory hypothesis of the CNS invasion of SARS-CoV-2. It reinforces the need to accumulate precise neurophysiological observations of COVID-19 patients worldwide and to aggregate multimodality screening of these patients also with clinical, radiological, biological, and neuropathological data.

## Author Contributions

GV collected the data and wrote the manuscript. RS, SV, PJ, and AR-T revised the manuscript. M-PO suggested and revised the manuscript. All authors contributed to the article and approved the submitted version.

## Conflict of Interest

The authors declare that the research was conducted in the absence of any commercial or financial relationships that could be construed as a potential conflict of interest.
